# *Notes from the Field*: Travel-Associated Measles in a Person Born Before 1957 — Pinellas County, Florida, 2019

**DOI:** 10.15585/mmwr.mm6938a6

**Published:** 2020-09-25

**Authors:** Kevin M. Baker, Abdiel E. Laureano-Rosario, Sharlene Edwards

**Affiliations:** 1 Florida Department of Health, Pinellas County.

Measles is a highly contagious respiratory disease; both locally acquired and travel-associated cases continue to occur across the United States (*1*). On April 19, 2019, the Pinellas County (Florida) Health Department (Pinellas CHD) Epidemiology Program was notified by a local hospital of a case of serologically confirmed measles in a man aged 72 years. The patient was evaluated in a hospital emergency department (ED) on April 15 with a 5-day history of fever, followed 2 days later by cough, and a maculopapular rash that started on his trunk on April 15. In the ED the patient experienced difficulty breathing and was admitted. In the hospital he received a diagnosis of pneumonia and subsequently developed sepsis and was transferred to the intensive care unit. Measles immunoglobulin (Ig) M and IgG were detected at commercial laboratories on April 15 and April 19, respectively. 

The patient’s wife reported that on April 12, she and her husband had returned from a month-long, multicountry trip to Asia. Their return trip included three flights on April 12, two of which landed at U.S. airports. After arriving home from the airport, the patient did not leave his house until seeking medical care at the ED on April 15. The patient’s wife reported that he had measles as a child, and therefore, had not been vaccinated against measles; however, no documentation was available to support that her husband had a childhood measles infection. Urine and nasopharyngeal swab specimens were collected on April 19 for measles polymerase chain reaction testing at the Florida Department of Health, Bureau of Public Health Laboratories; both specimens tested positive.

After measles was diagnosed on April 19, the Florida Department of Health notified the CDC Miami Quarantine Station that the patient had flown during his infectious period. CDC identified 31 exposed contacts on the inbound international flight and the domestic flight and notified three countries and four U.S. state health departments for follow-up. No secondary cases associated with the flights were identified. CDC also notified the destination country of the third flight.

The patient’s exposed close, personal contacts included his wife, his mother-in-law, and a friend; all were born before 1957 or had documented evidence of immunization or prior disease. While the patient was in the hospital ED, 432 other patients, visitors, and staff members were exposed. Line lists of patients, staff members, and other documented visitors were gathered in order to ascertain their measles immunity and begin the notification process. The hospital’s infection control program notified contacts exposed in the hospital by telephone and mail. Two patients and one visitor, identified more than 72 hours after measles exposure, were advised to receive postexposure prophylaxis with immune globulin; two of these persons received it within 6 days of their exposure. One infant contact did not receive postexposure prophylaxis within 6 days of exposure, and for this child, temperature was monitored daily for one measles incubation period (21 days) after the exposure (*2*).

The patient recovered and was discharged home from the hospital on April 27. None of his contacts developed measles, and no additional cases were identified through heightened surveillance, which included monitoring ED syndromic surveillance data in ESSENCE-FL* and notifying health care providers to immediately report suspected measles cases during the 21-day monitoring period.

The Advisory Committee on Immunization Practices recommends that adults without documentation of measles immunity who are traveling internationally receive 2 documented doses of measles, mumps, and rubella virus vaccine (*3*,*4*) before departure. A review of measles cases internationally imported into the United States during 2001–2016 found that 20 of 553 (4%) such cases occurred in persons born before 1957 (*5*). Persons born before 1957 are presumed to be immune because of the likelihood of having been infected with measles during childhood; however, the occurrence of measles in a person born before 1957 suggests that these cases can occur. Health care providers should consider measles in all persons who returned from international travel and are evaluated with febrile rash illness, regardless of age.

## Footnotes

* http://www.floridahealth.gov/diseases-and-conditions/disease-reporting-and-management/disease-reporting-and-surveillance/surveillance-systems.html.

## References

[1] Patel M, Lee AD, Redd SB, Increase in measles cases—United States, January 1–April 26, 2019. MMWR Morb Mortal Wkly Rep 2019;68:402–4. . 10.15585/mmwr.mm6817e1 31048672

[2] ACIP Adult Immunization Work Group. Bridges CB, Woods L, Coyne-Beasley T; CDC. Advisory Committee on Immunization Practices (ACIP) recommended immunization schedules for persons aged 0 through 18 years and adults aged 19 years and older—United States. MMWR Suppl 2013;62 (No. Suppl-1). 23364301

[3] McLean HQ, Fiebelkorn AP, Temte JL, Wallace GS; CDC. Prevention of measles, rubella, congenital rubella syndrome, and mumps, 2013: summary recommendations of the Advisory Committee on Immunization Practices (ACIP). MMWR Recomm Rep 2013 (No. RR-4). 23760231

[4] CDC. Measles prevention: recommendations of the Immunization Practices Advisory Committee (ACIP). MMWR Suppl 1989;38 (No. Suppl-9).

[5] Lee AD, Clemmons NS, Patel M, Gastañaduy PA. International importations of measles virus into the United States during the postelimination era, 2001–2016. J Infect Dis 2019;219:1616–23. 10.1093/infdis/jiy701 30535027 PMC6474820

